# Influence of subglottic secretion drainage on the microorganisms of ventilator associated pneumonia: A meta-analysis for subglottic secretion drainage

**DOI:** 10.1097/MD.0000000000011223

**Published:** 2018-07-13

**Authors:** Xu An Huang, Yan Ping Du, Bin Bin Fu, Liu Xia Li

**Affiliations:** a Medical College of Xiamen University; b Department of Respiratory Medicine, Zhongshan Hospital Xiamen University, Teaching Hospital of Fujian Medical University; c Fujian Medical University, Union Hospital, Zhongshan Hospital Xiamen University.

**Keywords:** meta-analysis, microorganisms, subglottic secretion drainage, ventilator associated pneumonia

## Abstract

**Background:**

The influence of the subglottic secretion drainage (SSD) on the microorganisms of ventilator associated pneumonia (VAP) is still unclear.

A meta-analysis focusing on the influence of the SSD on the microorganisms of VAP.

**Methods:**

A comprehensive search was conducted through the online studies of PubMed, Embase, Cochrane Library, Google scholar, CNKI (China National Knowledge Infrastructure), and VIPI (Database for Chinese Technical Periodicals) using specific search terms.

Included studies were randomized controlled trials (RCTs) that compare the microorganisms of VAP between SSD and standard endotracheal tube care in mechanically ventilated adults.

**Results:**

Nine RCTs were eligible. There was no significant difference in the rate of VAP caused by nonfermentative bacteria and enterobacteria between SSD group and control group (OR = 0.73, 95%CI, 0.53–1.01; *P = *.06). The episodes of VAP caused by Gram-positive cocci and *Haemophilus influenzae* organisms were lower in the SSD group (OR = 0.29, 95%CI, 0.18–0.48; P<0.00001). Less mean volume of SSD daily was observed in VAP group (OR = −16.97, 95%CI, −29.87–4.08; *P = *.010).

**Conclusion:**

We found SSD to be associated with significant decreases in VAP caused by Gram-positive cocci and *H influenzae* organisms but no significant differences in VAP caused by nonfermentative bacteria and enterobacteria. Less mean volume of SSD daily was observed in VAP group.

## 1 Introduction

Ventilator associated pneumonia (VAP) is very common nosocomial infection in the patients who require invasive mechanical ventilation.^[1]^ A number of prophylactic interventions advocated by various organizations include vigilant hand washing, early enteral feeding, sinusitis prophylaxis, head of the bed elevation, preservation of a normal gastric PH, selective digestive decontamination, conscientious oral care protocol, and subglottic secretion drainage (SSD).^[2]^

The concept of SSD is based on the hypothesis that decreasing aspiration of bacteria pooled above the cuff of the endotracheal tube into the lower respiratory tract reduces the risk of VAP. SSD using a specially designed endotracheal tube with a separate dorsal lumen which opens immediately above the endotrcheal cuff, has been developed to avoid the progression of subglottic secretions into the lower respiratory tract. SSD is widely used to prevent VAP. A survey from United States hospitals found that 55% of patients routinely use SSD.^[3]^ Guidelines from United States, Canada and Europe recommend SSD.^[4–6]^

Many meta-analyses suggest that SSD can reduce VAP rates but does not clearly decrease duration of mechanical ventilation, mortality or ICU length of stay,^[7–11]^ but the influence of the SSD on the microorganisms of VAP still required further data prove. For the limitations of prior meta-analyses, we undertook a meta-analysis focusing on the influence of the SSD on the microorganisms of VAP.

## 2 Methods

### 2.1 Search strategies

We searched all studies comparing SSD with standard endotracheal tubes and included at least microorganisms of ventilator associated pneumonia as an outcome. A comprehensive search was conducted through the online studies of PubMed, Embase, Cochrane Library, Google scholar, CNKI (China National Knowledge Infrastructure), and VIPI (Database for Chinese Technical Periodicals) from inception to August 2016. The following medical subject headings (MeSH) were searched: subglottic drainage, subglottic secretion, subglottic aspiration, ventilator associated pneumonia, and microorganisms. We traced the bibliographies of all retrieved trials and other related publications. We applied no language restriction. For our study is a meta-analysis so the ethical approval was not necessary.

### 2.2 Inclusion criteria/exclusion criteria

All randomized control trails (RCTs) comparing SSD with standard endotracheal tubes in the patients who require invasive mechanical ventilation were eligible. We also reviewed the references of all selected publications to ensure we had not missed any studies that could be included in our study. If there were more than one eligible publication from one publication from one author, the one with higher quality or the most recent publication date would be included. In addition, if the primary outcome was not the microorganisms of ventilator associated pneumonia, these studies were excluded.

### 2.3 Data extraction

Two reviewers independently evaluated the included studies and extracted data into RevMan. Any disagreement was resolved by discussion with a third reviewer. If still more data were required, communication through e-mail would be carried out with the authors.

### 2.4 Outcome measures

The primary endpoint of this meta-analysis was the VAP caused by nonfermentative bacteria and enterobacteria or VAP caused by Gram-positive cocci and *Haemophilus influenzae* organisms were measured from clinical features confirmed with endotracheal / bronchoscopically obtained cultures or by a good clinical response to antibiotic agents. The secondary endpoint was the mean volume of SSD daily between VAP group and non-VAP group was measured by accounted the volume of subglottic secretions aspirated every day.

### 2.5 Quality assessment

Firstly, all studies were assessed with the Jada Scale Scoring System.^[12]^ In which the best study quality is scored 5 points. Studies with a score ≥3 points were considered as high quality research and were enrolled. Secondly, studies were also classified by agreement of 2 authors as having a low risk of bias, an unclear risk of bias, or a high risk of bias based on the Cochrane tool. This tool takes into account random sequence generation, concealment of the allocation sequence, blinding of participants and personnel, blinding of outcome assessment, incomplete outcome, and selective reporting.

### 2.6 Statistical analysis

For each included study, odds ratio (OR) and 95% confidence intervals (CI) were calculated for dichotomous outcomes, and weighted mean differences (WMD) and 95% CI were calculated for continuous outcomes. Statistical heterogeneity was assessed using the *I*^2^ value ≤50% were considered as no statistical heterogeneity and used fixed-effects model to estimate the overall summary effect sizes. Otherwise, random-effects model was used a subgroup analysis or sensitivity analysis would be carried out. Risk of publication bias was assessed by visual inspection of funnel plots of effect sizes versus SE. We used Review Manager software (RevMan 5.3) and *P* value <.05 was considered as significant.

## 3 Results

### 3.1 Result of the search

**Figure 1:**
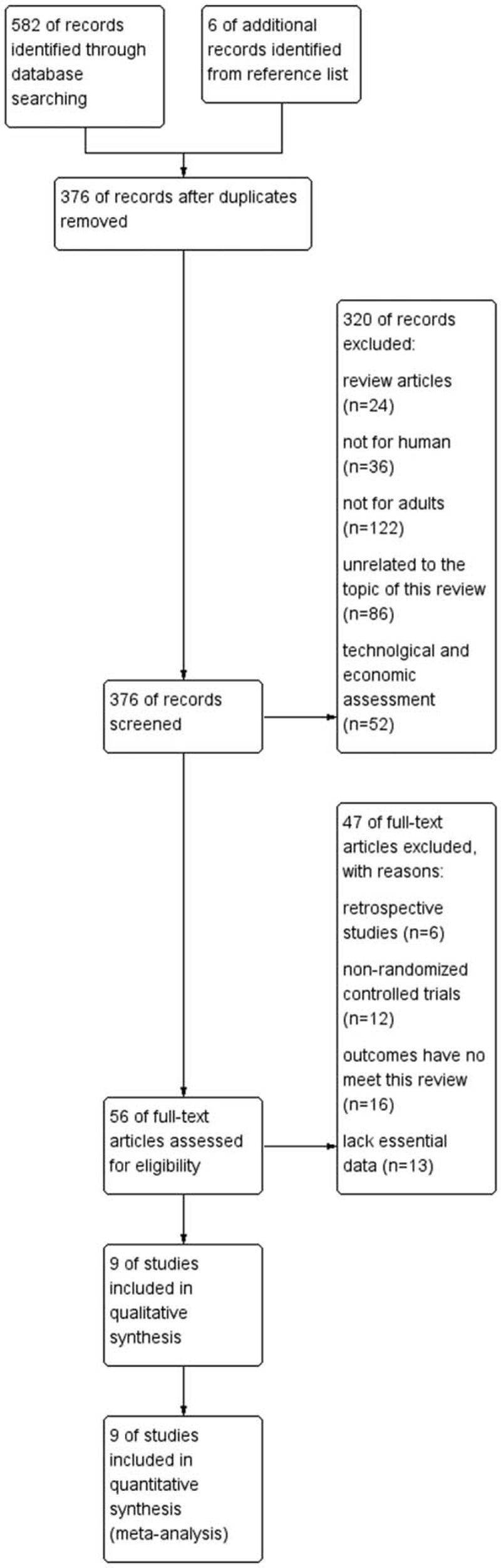
The graph shows a flow diagram of details search and exclusion criteria.

The 2016 update the search strategy that identified a total of 9 published RCTs were included in the final analysis^[13–21]^. The details of search and exclusion criteria are displayed in the flow diagram (Fig. 1).

### 3.2 Characteristics of selected studies

We identified 9 RCTs. All selected studies in our meta-analysis were published from 1995 to 2010. The selected study characteristics are summarized in Table 1.

**Table 1:**
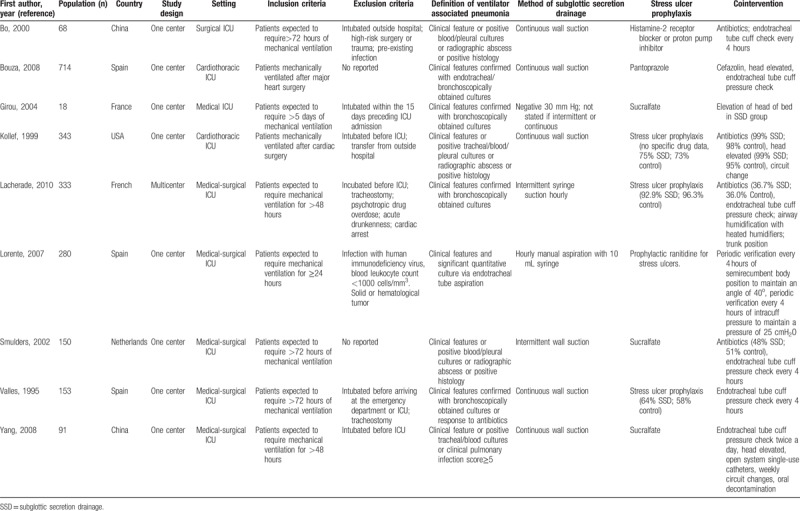
Characteristics of included studies.

### 3.3 Risk of bias

**Figure 2:**
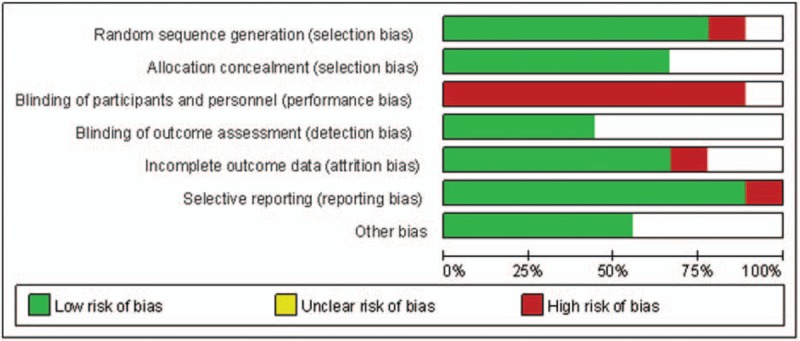
The graph shows a risk of bias graph.

**Figure 3:**
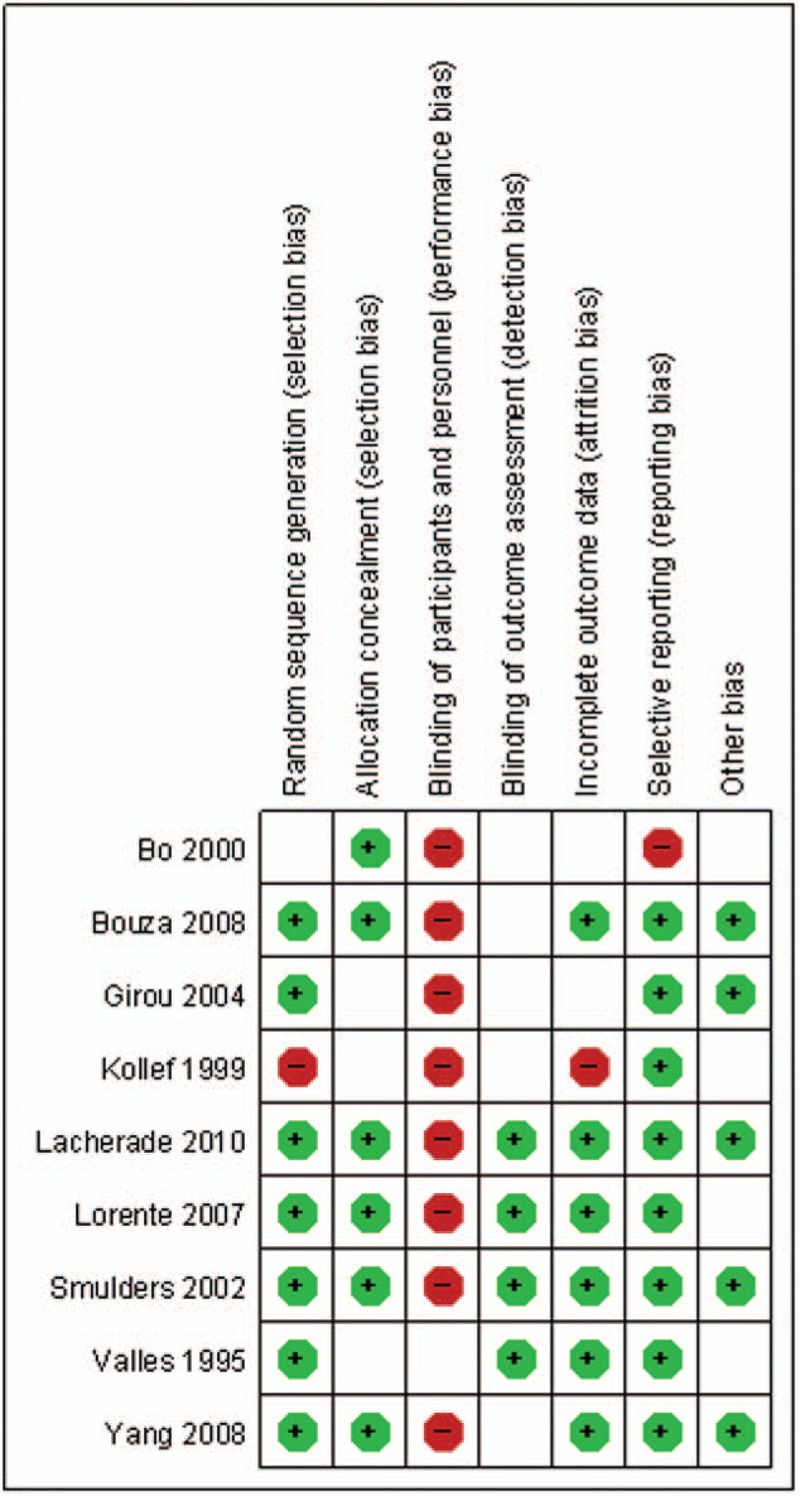
The graph shows a risk of bias summary.

The detailed risk of bias abut methodological quality of the included studies are elaborated and summarized, respectively, in Figure 2 and Figure 3.

### 3.4 Meta-analysis results

#### 3.4.1 The primary endpoint

**Figure 4:**
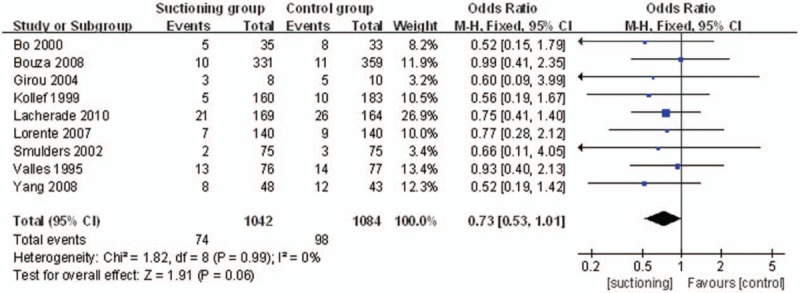
The graph shows a forest plot of relative risk with confidence interval for VAP caused by nonfermentative bacteria and enterobacteria. VAP = ventilator associated pneumonia.

The primary endpoint “the episodes of VAP caused by nonfermentative bacteria and enterobacteria” was reported in all 9 studies. A total of 1042 patients in the SSD group and 1084 patients in the control group were available to compare the episodes of VAP. The pooled results showed that there was no significant difference in the rate of VAP caused by nonfermentative bacteria and enterobacteria between SSD group and control group (OR = 0.73, 95%CI, 0.53–1.01; *P = *.06) (Fig. 4).

**Figure 5:**
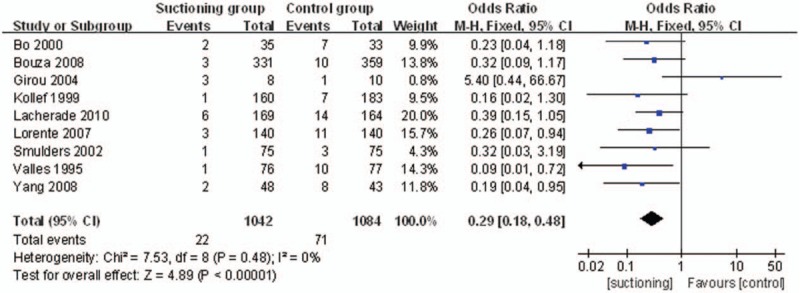
The graph shows a forest plot of relative risk with confidence interval for VAP caused by Gram-positive cocci and *H influenzae* organisms. VAP = ventilator associated pneumonia.

The endpoint “the episodes of VAP caused by Gram-positive cocci and *H influenzae* organisms” was reported in all 9 studies. A total of 1042 patients in the SSD group and 1084 patients in the control group were available to compare the episodes of VAP. The episodes of VAP caused by Gram-positive cocci and *H influenzae* organisms were lower in the SSD group. (OR = 0.29, 95%CI, 0.18–0.48; *P*<.00001) (Fig. 5).

#### 3.4.2 The secondary endpoint

**Figure 6:**
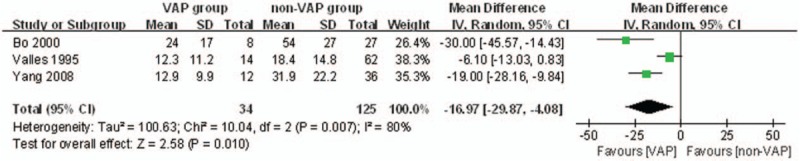
The graph shows a forest plot of relative risk with confidence interval for the mean volume of SSD daily between VAP group and non-VAP group. SSD = subglottic secretion drainage, VAP = ventilator associated pneumonia.

The secondary endpoint “the mean volume of SSD daily between VAP group and non-VAP group” was reported in 3 studies. A total of 34 patients in the VAP group and 125 patients in the non-VAP group were available to compare the mean volume of SSD daily. Due to the high heterogeneity (*P = *.007, *I*^2^ = 80%), random-effects model was adopted to pool the data. Less mean volume of SSD daily was observed in VAP group (OR = −16.97, 95%CI, −29.87–4.08; *P = *.010) (Fig. 6).

## 4 Discussion

The prevention of VAP is important for its impact on patient outcomes, costs and need for additional antibiotic. Many prevention measures are currently available. The combined use of SSD and continuous control of endotracheal tube cuff pressure reduced the incidence of ventilator-associated respiratory infection and saved health care costs.^[22]^ We performed a meta-analysis to determine the effectiveness of SSD to prevent VAP in patients undergoing mechanical ventilation.

We found SSD to be associated with significant decreases in VAP caused by Gram-positive cocci and *H influenzae* organisms but no significant differences in VAP caused by nonfermentative bacteria and enterobacteria. Safdari et al^[23]^ found a significant reduction in the incidence of early-onset VAP by using SSD with inspiratory pause maneuver among intubated patients. We know that early onset VAP which is related to micro-inhalation is more often caused by Gram-positive cocci and *H influenzae*. Meanwhile Gram-positive cocci and *H influenzae* are also frequently retrieved in head trauma patients and severe subarachnoid hemorrhage patients. The risk of micro-inhalation due to unconsciousness is often advocated. We suspect VAP caused by Gram-positive cocci and *H influenzae* organisms is more often associated with microinhalation, so it can be decreased by SSD. But the increased use of antibiotics, which may contribute to nonfermentative bacteria and enterobacteria infection in VAP patients, so the VAP caused by nonfermentative bacteria and enterobacteria can not be decreased by SSD.

The minimum inoculum needed for development of Gram-positive cooci or *H influenzae* pneumonia is higher than the inoculum needed for nonfermentative bacteria and enterobacteria pneumonias. This could explain the decrease of Gram-positive cooci or *H influenzae* pneumonias in SSD group. We also found the volume of subglottic secretions aspirated in patients with pneumonia was lower than that in patients without pneumonia. This may indicate an increase in the volume of secretions aspirated due to the reduction in the effectiveness of subglottic suction. This finding also told us the importance of maintaining adequate cuff pressure to prevent microaspirations to the bronchial tract. If the cuff pressure do not maintain adequate it will reduce the volume of subglottic secretions, and patients have more chance to get VAP. And if we do not aspirate subglottic secretion effectively it can cause the volume of subglottic secretions aspirated decrease, and also increase VAP rate.

In Lacherade 2010 study it is found that more patients got postextubation laryngeal dyspnea in SSD group than control group. In Girou 2004 study, 2 patients developed laryngeal edema immediately after extubation in SSD group, but no patient developed laryngeal edema in control group. It was reported that 40% of the patients under continuous suctioning developed laryngeal edema and tracheal mucosal erythema, haemorrhage or necrosis.^[24]^ Many studies chose intermittent instead of continuous subglottic suctioning because of the risk of damaging the tracheal wall. But Frost et al^[10]^ did not find significant adverse events in their meta-analysis from using continuous suction. Berra and Girou have found some adverse effects on the tracheal wall from continuous suction not only in animals but also in humans.^[16,25]^ So subglottic secretion though manual suction may be better.

One limitation is that in Lorente 2007 study, we were not able to discriminate the independent influence of SSD and polyurethane cuff in the microorganisms of ventilator associated pneumonia. The other limitation is that not all included studies diagnosis of VAP used quantitative cultures of lower respiratory secretion though bronchoscope but just used tracheal secretion culture. Study form Browne has found that use of tracheal secretion culture to diagnosis VAP is as sensitive as bronchoscope.^[26]^

## 5 Conclusions

SSD is associated with significant reductions in VAP caused by Gram-positive cocci and *H influenzae* organisms but no significant differences in VAP caused by nonfermentative bacteria and enterobacteria. Less mean volume of SSD daily was observed in VAP group.

## Author contributions

**Conceptualization:** Xu An Huang, Yan Ping Du.

**Data curation:** Xu An Huang, Yan Ping Du.

**Formal analysis:** Yan Ping Du, Liu Xia Li.

**Investigation:** Yan Ping Du.

**Methodology:** Xu An Huang, Yan Ping Du, Liu Xia Li.

**Software:** Bin Bin Fu, Xu An Huang.

**Writing – review & editing:** Xu An Huang, Yan Ping Du.
